# rSIG combined with NLR in the prognostic assessment of patients with multiple injuries

**DOI:** 10.1515/med-2025-1161

**Published:** 2025-04-08

**Authors:** Dan Wei, Xing Liu, Yanlai Gu, Qiuhong Fu, Hua Tang

**Affiliations:** Department of Emergency, Shenzhen Longhua District Center Hospital, Shenzhen, Guangdong, 518110, P. R. China; Department of Emergency, The Second People’s Hospital of Futian District Shenzhen, Shenzhen, Guangdong, 518049, P. R. China; Department of Emergency, Shenzhen Longhua District Center Hospital, 187 Guanlan Dadao West, Longhua District, Shenzhen, Guangdong, 518110, P. R. China

**Keywords:** multiple injuries, rSIG, NLR, ISS, prognosis

## Abstract

**Objective:**

To investigate the significance of the reverse shock index multiplied by the Glasgow Coma Scale score (rSIG) and the neutrophil–lymphocyte ratio (NLR) in the prediction of prognosis in patients with multiple injuries.

**Methods:**

The clinical data of 142 patients with multiple injuries admitted to the Emergency Department of Shenzhen Longhua District Central Hospital between January 2019 and December 2022 were retrospectively analyzed. Subjects were divided into the survival group (*n* = 102) and the deceased group (*n* = 40) based on their survival status at 28 days after injury. We subsequently analyzed the intergroup differences in blood test results, rSIG, and NLR, as well as the relationship between rSIG and NLR. The predictive value of rSIG, NLR, and both combined in determining the prognosis of patients with multiple injuries was explored by plotting the receiver operator characteristic (ROC) curve. Based on the optimal cut-point value of the ROC curves, subjects were divided into groups with rSIG ≤ 7.75 (22 patients) and rSIG > 7.75 (120 patients), as well as groups with NLR ≤ 10.36 (104 patients) and NLR > 10.36 (38 patients), and the 28-day mortality rate was compared between the groups.

**Results:**

A total of 142 patients were enrolled. The rSIG of the survival group (*n* = 102) was significantly greater (15.7 ± 4.8) than that of the deceased group (*n* = 40, 6.2 ± 2.9), (*t* = 14.307, *p* < 0.001). The NLR of the survival group was higher than that of the deceased group, but the difference was not statistically significant (*p* > 0.05). The area under the curve (AUC) of the ROC of NLR was significantly greater than that of rSIG (0.922 vs 0.54) (*Z* = −7.881, *p* < 0.001). The AUC for NLR was also statistically greater than that of the combination of rSIG and NLR (0.963 vs 0.54) (*Z* = −8.378, *p* < 0.001). The AUC of the combination of rSIG and NLR was significantly greater than that of rSIG (0.844 vs 0.540) (*Z* = 2.239, *p* = 0.025). The 28-day mortality rate of patients in the rSIG ≤ 7.75 group was also significantly greater than that of patients in the rSIG > 7.75 group (10.0%) (*p* < 0.05). Finally, the 28-day mortality rate in the group with an NLR ≤ 10.36 was lower than that in the group with an NLR > 10.36 (*p* < 0.05). Pearson correlation analysis showed that the correlation coefficient between rSIG and NLR was *r* = 0.13, which did not reach statistical significance (*p* = 0.12).

**Conclusion:**

NLR, rSIG, and the combination of the two are all valuable in predicting the prognosis of patients with multiple injuries (all AUC > 0.5). However, the predictive capacity of NLR was better than either rSIG alone or both combined. These findings may serve as references in guiding the treatment of patients with multiple injuries in clinical practice.

## 1 Introduction

Approximately 5 million people die from violence or trauma worldwide every year, accounting for a significant portion of the world’s mortality burden [1]. Among trauma patients, the difficulty of treatment is increased for those with multiple injuries in several body parts due to the complexity of their conditions [2]. In the treatment of patients with multiple injuries, early assessment of the severity of the patient’s injuries is required to allow timely transport of the patient or necessary treatment measures to reduce the mortality and disability rates and improve patient prognosis [3,4]. The Injury Severity Score (ISS) is commonly used in clinical practice but is not without its limitations. The ISS is a trauma assessment system from the anatomical perspective, which does not take into account the impact of physiological factors, such as age and physical fitness, on trauma and prognosis. The shock index (SI) is a more sensitive indicator of the level of shock than traditional vital signs, while the Glasgow Coma Scale score (GCS) shows a strong predictive ability for the prognosis of trauma patients [5,6,7]. Based on this information, some researchers [8] have proposed a new parameter: the reverse shock index multiplied by the Glasgow Coma Scale score (rSIG), which is derived by dividing the SI from the GCS. Studies have shown that the rSIG may serve as a good reference for the prediction of the mortality risk of trauma patients [9,10].

The neutrophil–lymphocyte ratio (NLR) is a composite inflammatory marker that has emerged in recent years and is currently recognized as a marker that allows rapid assessment of the patient’s immune and inflammatory status [11]. Compared with other inflammatory markers such as the C-reactive protein, NLR has its own characteristics. NLR integrates two different but complementary immune pathways and reflects the level of the body’s stress response through lymphocyte counts. Moreover, the deterioration of the systemic inflammatory response is reflected through the neutrophil counts. A lower ratio indicates a higher level of stress [12].

Based on the presented results, this study aimed to explore the utility of rSIG and NLR in predicting the prognosis of patients with multiple injuries by retrospectively analyzing the clinical data of patients with multiple injuries, which is reported as follows.

## 2 Data and methods

### 2.1 General data

Retrospective analysis was performed to collect the clinical data of patients with multiple injuries admitted to the emergency room of Shenzhen Longhua District Central Hospital from January 2019 to December 2022. The analysis included a total of 142 patients. Inclusion criteria: (1) patients who were injured for the first time; (2) time from injury to admission < 24 h; (3) patients aged > 14 years; (4) patients with multiple injuries diagnosed by relevant testing or physical examination; and (5) patients with complete clinical records and medical history. Exclusion criteria: (1) patients aged < 14 years old; (2) patients who were dead at admission; and (3) patients with a previous history of underlying diseases of the vital organs, such as the heart, brain, and kidney, or malignant tumors.

### 2.2 Methods

The patient’s basic information, including gender, age, vital signs, time from injury to admission, and cause of injury, was recorded through the electronic medical record system, and the corresponding scoring was calculated, including the rSIG, GCS, and ISS. Blood samples were collected for routine blood, biochemical, and coagulation function testing. The automatic modular blood and body fluid analyzer (model: BC-7500) produced by Mindray was used for routine blood testing, the automatic biochemical analyzer (model: VITROS-5600) produced by Johnson & Johnson was used for biochemical testing, and the automatic coagulation analyzer (model: CS-5100) produced by Sysmex Corporation of Japan was used for coagulation-related tests.

### 2.3 Statistical analysis

SPSS 26.0 statistical software was used to analyze the collected data. The Kolmogorov–Smirnov test was performed to test the normality of measurement data. Measures that conformed to normal distribution are expressed as the mean *±* standard deviation (*x ± s*), and intergroup comparisons for these data were conducted using a random-design independent samples *t*-test. Measures that were not normally distributed are expressed as the median (interquartile spacing) [M (Q1,Q3)], and intergroup comparisons were made using the Mann–Whitney *U* test. Count data are expressed as the frequency (%), and intergroup comparisons were made using the *χ* ^2^ test. ROC curves of rSIG and NLR were produced using MedCalc 15.2.2. The DeLong nonparametric approach was adopted to compare the respective AUC of the ROC curves and to analyze the significance of rSIG and NLR in determining the prognosis of patients with multiple injuries. The predictive value is evaluated by the area under the ROC curve. If the AUC value is between 0.85 and 0.95, the prediction effect is good; If the AUC value is between 0.7 and 0.85, the prediction effect is average; If the AUC value is between 0.5 and 0.7, the effect is low; If the AUC value is less than 0.5, it has no predictive value. The Pearson method was used to analyze the correlation between rSIG and NLR. A *p*-value of less than 0.05 was considered statistically significant.


**Ethics approval:** This study was approved by the Medical Ethics Committee of Shenzhen Longhua District Central Hospital. No: 2024-002-01.

## 3 Results

### 3.1 General data

**Figure 1:**
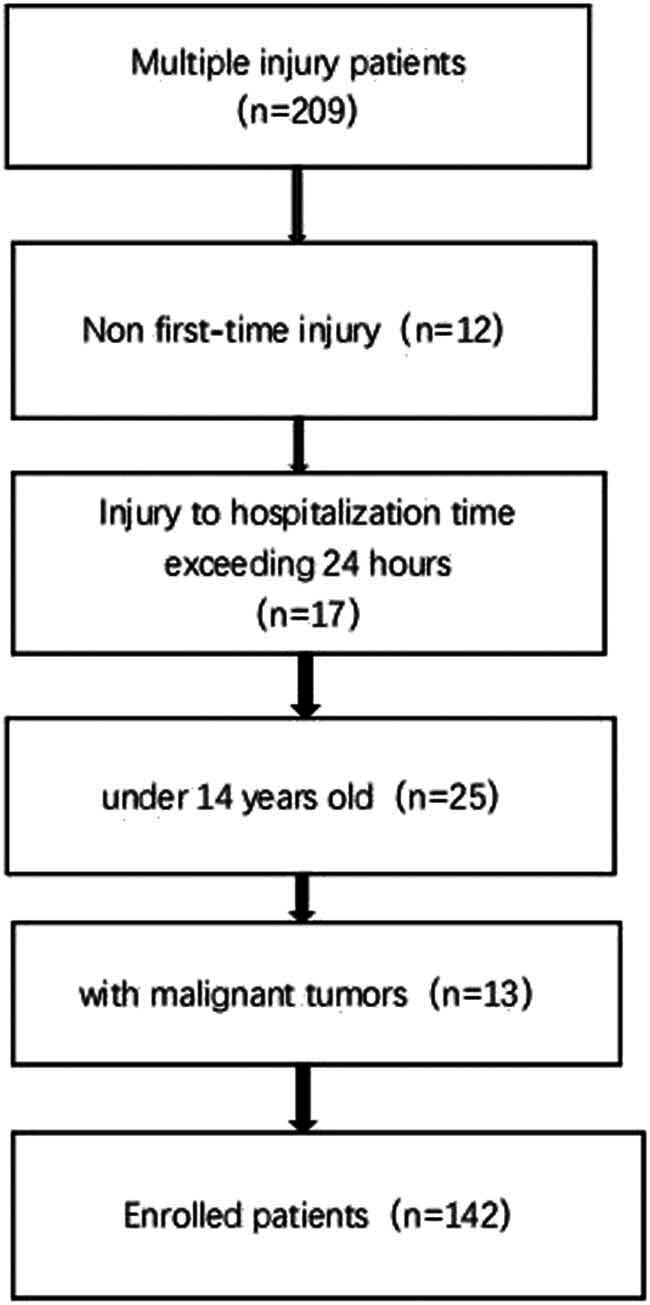
Flow chart figure.

A total of 142 patients were enrolled as shown in Figure 1. Among them, there were 116 males and 26 females. According to the survival outcome, there were 102 cases in the survival group and 40 cases in the death group. The top three causes of injury were as follows: 65 cases of car accident injury (45.77%), 37 cases of high-altitude falling injury (26.05%), and 20 cases of falling injury (14.08%). The remaining causes of injury included knife stab wounds, blunt injuries, and so on. A comparison of the rest of the general patient data between the two groups is shown in Table 1.

**Table 1:** Comparison of the general data of patients with multiple injuries in the survival and deceased groups

Observational indicators	Survival group (*N* = 102)	Deceased group (*N* = 40)	Statistic	*p*-Value
Age [M (Q1,Q3)]	36.0(28.0,49.8)	42.5(30.0,51.0)	*W* = 1816.000	*p* = 0.310
M/F (no. of subjects)	84/18	32/8	*χ* ^2^ = 0.007	*p* = 0.932
White blood cell [M (Q1,Q3)]	8.7(4.3,13.8)	12.2(6.7,15.4)	*W* = 1618.000	*p* = 0.056
PLT [M (Q1,Q3)]	237.0(175.2,312.5)	220.5(116.5,296.2)	*W* = 2358.000	*p* = 0.150
Total protein [M (Q1,Q3)]	70.0(57.0,79.0)	61.0(48.8,75.0)	*W* = 2406.000	*p* = 0.097
Neutrophils [M (Q1,Q3)]	9.6(5.2,13.6)	10.2(5.9,15.2)	*W* = 1880.000	*p* = 0.469
Lymphocytes [M (Q1,Q3)]	2.8(1.8,3.2)	2.2(1.5,3.1)	*W* = 2337.000	*p* = 0.179
NLR [M (Q1,Q3)]	4.0(2.1,7.0)	3.8(1.8,5.6)	*W* = 2204.500	*p* = 0.457
Alanine transaminase [M (Q1,Q3)]	148.5(103.0,204.8)	151.0(100.5,211.5)	*W* = 1993.000	*p* = 0.833
Aspartate transaminase [M (Q1,Q3)]	69.0(30.8,100.2)	48.5(33.2,86.2)	*W* = 2290.000	*p* = 0.258
Creatinine [M (Q1,Q3)]	105.5(80.2,142.8)	98.5(72.8,140.2)	*W* = 2200.500	*p* = 0.468
D-D (μg/ml) [M (Q1,Q3)]	7.4(4.3,11.3)	28.1(18.3,33.9)	*W* = 254.000	*p* < 0.001
ISS [M (Q1,Q3)]	26.0(20.0,34.0)	41.0(34.8,50.0)	*W* = 641.500	*p* < 0.001
Heart rate [M (Q1,Q3)]	114.0(105.0,124.0)	132.0(124.8,137.0)	*W* = 647.500	*p* < 0.001
Respiratory rate [M (Q1,Q3)]	22.0(20.0,24.0)	29.0(25.0,31.0)	*W* = 1086.500	*p* < 0.001
Systolic pressure [M (Q1,Q3)]	128.5(105.5,139.0)	102.0(84.0,152.2)	*W* = 2369.500	*p* = 0.136
Shock index (SI) [M (Q1,Q3)]	0.9(0.8,1.1)	1.2(0.8,1.7)	*W* = 1208.000	*p* < 0.001
GCS [M (Q1,Q3)]	15.0(14.0,15.0)	6.5(5.0,8.0)	*W* = 3949.000	*p* < 0.001
Hemoglobin (±s)	129.7 ± 23.2	116.3 ± 29.5	*t* = 2.585	*p* = 0.012
Diastolic blood pressure (±s)	77.7 ± 15.6	77.9 ± 28.5	*t* = −0.038	*p* = 0.970
Mean arterial pressure (±s)	93.1 ± 16.7	90.2 ± 30.2	*t* = 0.570	*p* = 0.571
rSIG (±s)	15.7 ± 4.8	6.2 ± 2.9	*t* = 14.307	*p* < 0.001

Note: M: median; Q1,Q3: interquartile spacing; s: standard deviation; GCS: Glasgow Coma Scale score; ISS: Injury Severity Score; NLR: neutrophil-lymphocyte ratio; rSIG: the reverse shock index multiplied by Glasgow Coma Scale score; PLT: blood platelet; D-D: D-Dimer.

### 3.2 Pearson correlation analysis

**Figure 2:**
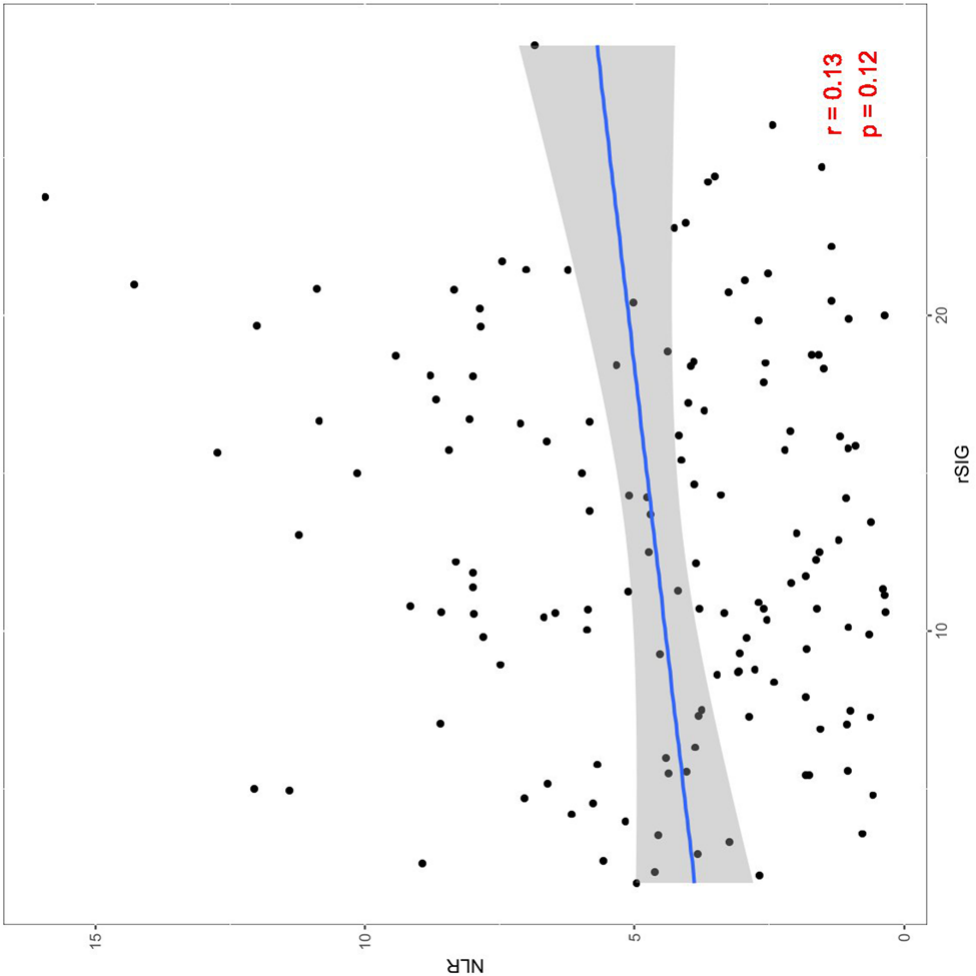
Pearson correlation analysis between rSIG and NLR levels.

Pearson correlation analysis between rSIG and NLR showed that the correlation coefficient in patients with multiple injuries was *r* = 0.13, *p* = 0.12, which was not statistically significant (Figure 2).

### 3.3 Values of rSIG, NLR, and combination of the two in predicting the prognosis of patients with multiple injuries

**Figure 3:**
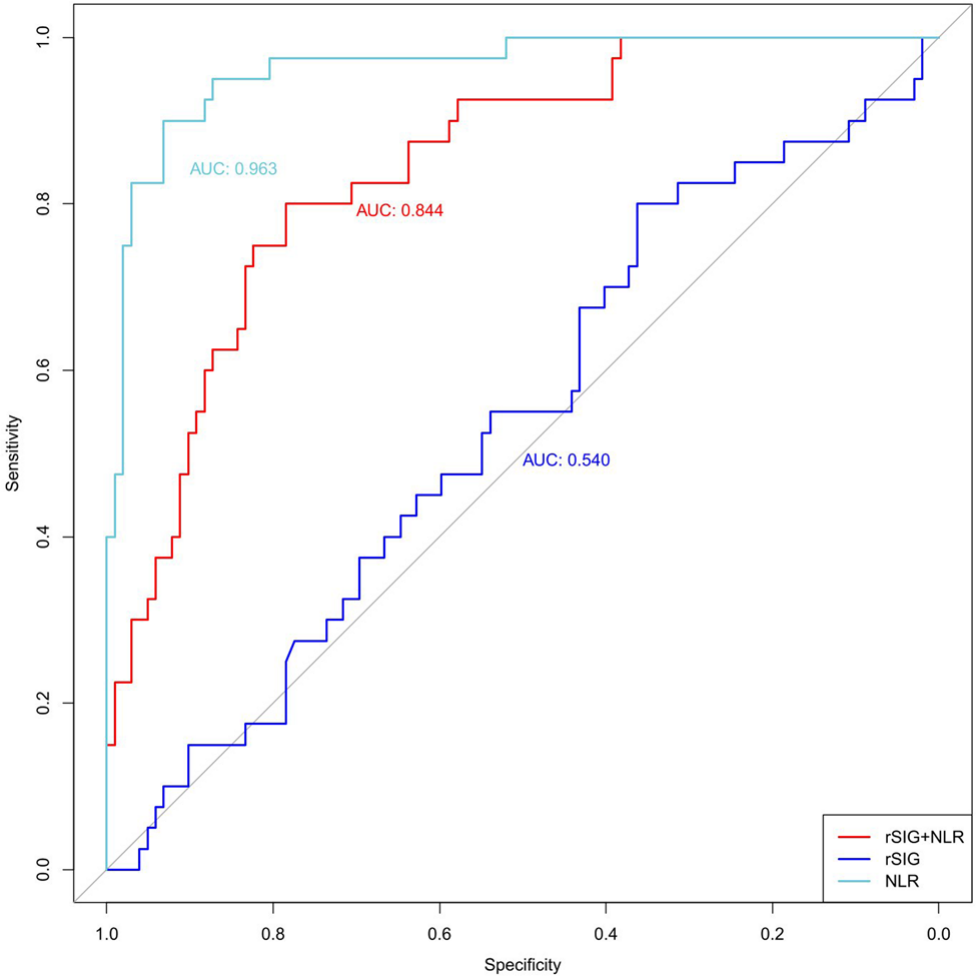
AUC for rSIG, NLR, and the combination of the two indicators. rSIG: the reverse shock index multiplied by the Glasgow Coma Scale score. NLR: neutrophil-lymphocyte ratio.

Receiver operating characteristic (ROC) curves were generated, and areas under the ROC curve (AUCs) were compared to explore the capacity of rSIG and NLR alone and in combination for predicting the prognosis of patients with multiple injuries. All three indicators showed a certain value in predicting the 28-day mortality in patients with multiple injuries (AUC > 0.5 for all indicators). The AUC of NLR was greater than that of rSIG (0.922 vs 0.54) (*Z* = −7.881, *p* < 0.001). The AUC of NLR was also greater than that of the combination of rSIG and NLR (0.963 vs 0.54) (*Z* = −8.378, *p* < 0.001). The AUC of the combination of rSIG and NLR was significantly greater than that of rSIG (0.844 vs 0.540) (*Z* = 2.239, *p* = 0.025). The details are shown in Figure 3 and Tables 2 and 3.

**Table 2:** Comparison of the assessment capacity of rSIG, NLR, and the combination of the two indicators

Predictive indicator	Cut-point value	Sensitivity	Specificity	AUC	Error	95% CI
NLR	9.61	90.00%	93.14%	0.963	0.008	(0.866, 0.977)
rSIG	5.78	80.00%	36.27%	0.540	0.027	(0.436, 0.645)
Combination of NLR and rSIG				0.844	0.014	(0.933, 0.994)

Note: NLR: neutrophil-lymphocyte ratio; rSIG: the reverse shock index multiplied by the Glasgow Coma Scale score.

**Table 3:** Comparison between rSIG, NLR, and the combination of rSIG and NLR

Compared indicators	*Z*-value	*p*-Value
NLR vs rSIG	−7.881	<0.001
NLR vs combined NLR and rSIG	−8.378	<0.001
rSIG vs combined NLR and rSIG	2.2397	0.025

Note: NLR: neutrophil-lymphocyte ratio; rSIG: the reverse shock index multiplied by the Glasgow Coma Scale score.

### 3.4 Comparison of mortality in patients with multiple injuries in different rSIG groups

The results of ROC curve analysis showed that the optimal cut-point value for rSIG was 7.75. Accordingly, the patients were divided into two groups: rSIG ≤ 7.75 (22 subjects) and rSIG > 7.75 (120 subjects). The difference in NLR levels between the two groups was statistically significant (*p* = 0.0253, <0.05), with the rSIG ≤ 7.75 group having significantly higher NLR levels than the rSIG > 7.75 group. The 28-day mortality rate in the rSIG ≤ 7.75 group was significantly lower than that in the rSIG > 7.75 group, as shown in Table 4.

**Table 4:** Comparison of NLR and 28-day mortality in patients with multiple injuries in different rSIG groups

Group	NLR	28-day mortality [No. of subjects (%)]
Group with rSIG ≤ 7.75 (*n* = 22)	4.791(2.359,6.888)	10(100)
Group with rSIG > 7.75 (*n* = 120)	2.529(1.223,3.681)	30(22.7)
Statistic	*w* = 941.0	*χ* ^2^ = 23.746
*p*-Value	*p* = 0.0253	<0.001

Note: NLR: neutrophil-lymphocyte ratio; rSIG: the reverse shock index multiplied by the Glasgow Coma Scale score.

### 3.5 Comparison of mortality in patients with multiple injuries in groups with different NLR levels

The results of ROC curve analysis showed that the optimal cut-point value for NLR was 10.36. Patients were accordingly divided into two groups: NLR ≤ 10.36 (133 subjects) and NLR > 10.36 (9 subjects). The 28-day mortality rate in the group with an NLR ≤ 10.36 was lower than that in the group with an NLR > 10.36 (*p* < 0.05). The rSIG in the group with an NLR ≤ 10.36 was higher than that in the group with an NLR > 10.36 (*p* < 0.05) (see Table 5).

**Table 5:** Comparison of rSIG and 28-day mortality in patients with multiple injuries grouped by different NLR levels

Group	rSIG	28-day mortality rate (%)
NLR ≤ 10.36 (*n* = 133)	27.984(24.264,31.732)	37(27.8)
NLR > 10.36 (*n* = 9)	16.926(11.494,21.803)	3(33.3)
Statistic	*W* = 1025.0	<0.001^*^
*p*-Value	*p* < 0.001	1

Note: *Minimum expectation was 2.54, and Yate’s correction for continuity was performed; NLR: neutrophil-lymphocyte ratio; rSIG: the reverse shock index multiplied by the Glasgow Coma Scale score.

## 4 Discussion

Trauma is one of the leading causes of death in the young population worldwide, with multiple injuries being a common critical condition encountered in emergency departments [13]. Early diagnosis and treatment are crucial for multiple injuries, while early identification of critically ill patients and timely transfer to high-level trauma centers may reduce the mortality rate and improve the prognosis of patients, as well as improve the utilization of medical resources [14,15,16]. In the present study, the prognostic assessment capacity of NLR, rSIG, and the combination of the two for patients with multiple injuries were calculated. NLR was found to have a favorable predictive capacity for the prognosis of patients with multiple injuries, while the predictive capacity of rSIG was relatively low. The predictive capacity of the combination of NLR and rSIG was better than that of rSIG alone but worse than that of NLR alone for patients with multiple injuries. The cut-off values for rSIG ≤ 7.75 and NLR > 10.36 were associated with a higher 28-day mortality rate. The correlation coefficient between rSIG and NLR did not reach statistical significance.

The reverse shock index has been shown to be more sensitive at determining the prognosis of trauma patients than SI [17,18]. Relevant studies in China and abroad have reported that rSIG had favorable accuracy in determining the prognosis of trauma patients, with the rSIG of the survival group being significantly higher than that of the deceased group [19,20]. In this study, rSIG was higher in the survival group than in the deceased group, which was consistent with previous studies, suggesting that the prognosis of trauma patients may be determined using rSIG.

Wu et al. [12] first reported the correlation between NLR and the prognosis of critically ill patients, showing an increase in neutrophils and a decrease in lymphocytes (resulting in an increase in NLR), 4–8 h after the body undergoes a severe attack such as a serious infection or a trauma. NLR, a simple inflammation assessment indicator that is easy to measure, can be calculated directly from the patient’s neutrophil and lymphocyte counts without the need for any other invasive testing and has thus been widely applied in the analysis of the severity and adverse prognosis of acute and chronic diseases such as pancreatitis, renal failure, coronary artery disease, and tumors [9,21–25].

NLR has also been used in the prediction of the prognosis of patients undergoing abdominal surgery, critical trauma, and acute kidney injury [26–29]. However, there have been few reports on the use of NLR in patients with multiple injuries. Heffernan et al. noted that neutrophilia and lymphopenia were also commonly observed in trauma patients [30]. Multiple injuries are characterized by rapid changes in injury conditions, high morbidity and mortality rates, and vulnerability to infections. Multiple injuries may activate the systemic inflammatory system, resulting in the release of a large number of inflammatory factors, which may elicit the systemic inflammatory response syndrome [31]. During the acute phase of inflammation, a large number of neutrophils are activated and migrate extensively throughout the body [32]. Meanwhile, trauma and blood loss can inhibit the activity of T-lymphocytes and B-lymphocytes, which in turn decreases the number of CD4+ T-cells and natural killer cells, leading to low immunity of the body, thereby greatly increasing the possibility of the patient suffering from complications of severe sepsis and multi-organ system failure [33–35]. Hence, during the early stages of multiple injuries, these outcomes would correspond to an increased number of neutrophils, a decreased number of lymphocytes, and a corresponding increase in the NLR level. Neutrophils and lymphocytes play an important role in the immune response to infectious inflammation. In this study, the results suggested that NLR levels were higher in the survival group than in the deceased group, but the difference was not statistically significant. This was different from the results of other studies [36], which may be explained by both the small sample size, and the fact that the subjects included in this study had multiple injuries. Moreover, as our hospital is a primary healthcare institution, there may be deficiencies in the capacity to treat trauma, which has led to a higher mortality rate of patients with multiple injuries and thus differences in the study findings. This suggested that when treating patients with multiple injuries in clinical practice, we can focus on the changes in NLR levels, as this index may be more helpful in the prognostic assessment of patients.

## 5 Limitations

This study has several limitations. The sample size is relatively small, which may restrict the generalizability of our results. The retrospective design introduces potential biases, and the single-center setting may limit the diversity of the patient population. Additionally, the timing of blood collection and concurrent interventions could influence rSIG and NLR measurements, which were not accounted for in this analysis. The non-significant correlation between rSIG and NLR also suggests the need for further exploration of their relationship. Lastly, the study did not evaluate the practicality or cost-effectiveness of implementing these markers in clinical practice. Future research with larger, multicenter cohorts is needed to address these limitations.

## 6 Conclusions

Patients with multiple injuries have critical and complex conditions that progress rapidly and require active treatment. The rSIG index and NLR are associated with the prognosis of patients with multiple trauma. Dynamic evaluation of the rSIG index and monitoring NLR level in patients with multiple trauma can evaluate the prognosis of patients with multiple trauma and have certain guiding significance for the treatment of multiple trauma in emergency. In future, the relationship between rSIG and the prognosis of patients with trauma can be further discussed; at the same time, there is also a need for more samples or more center joint research for deep research.
